# Basic human values during the COVID-19 outbreak, perceived threat and their relationships with compliance with movement restrictions and social distancing

**DOI:** 10.1371/journal.pone.0253430

**Published:** 2021-06-18

**Authors:** Eric Bonetto, Guillaume Dezecache, Armelle Nugier, Marion Inigo, Jean-Denis Mathias, Sylvie Huet, Nicolas Pellerin, Maya Corman, Pierre Bertrand, Eric Raufaste, Michel Streith, Serge Guimond, Roxane de la Sablonnière, Michael Dambrun

**Affiliations:** 1 Université Clermont Auvergne, CNRS, LAPSCO, Clermont-Ferrand, France; 2 Aix-Marseille University, Aix-en-Provence, France; 3 CINBIOSE Research Group, UQAM, Montréal, Québec, Canada; 4 Université Clermont Auvergne, INRAE, UR LISC, Centre de Clermont-Ferrand, Aubière, France; 5 Université Toulouse 2 Jean Jaurès, CNRS, CLLE, Toulouse, France; 6 Université de Montréal, Montréal, Québec, Canada; Middlesex University, UNITED KINGDOM

## Abstract

This study examines the evolution of Schwartz’s Basic Human Values during the COVID-19 outbreak, and their relationships with perceived threat, compliance with movement restrictions and social distancing. An online questionnaire was administered to a heterogeneous sample of French citizens (*N* = 1025) during the first French lockdown related to the outbreak. Results revealed a significant evolution of values; the conservation value was higher during the outbreak than usual, and both self-enhancement and openness-to-change values were lower during the COVID-19 outbreak than usual. Conservation and perceived threat during the outbreak were robustly and positively related to both compliance with movement restrictions and social distancing. Conservation during the outbreak emerged as a significant partial mediator of the relationship between perceived threat and outcomes (i.e., compliance with movement restrictions and social distancing). Implications of these results for the malleability of values and the COVID-19 modelling are discussed.

## Introduction

The current COVID-19 pandemic is undeniably the most serious sanitary crisis the world has known since the Great Influenza of 1918. When we are reminded about the great, destructive and long-term impact the Great Influenza has had on societies (not only in terms of fatalities, but also in regards to long-lasting political and financial consequences [1–3]), the race for obtaining a solution to put an end to this pandemic is becoming increasingly urgent. Despite the long history of our interactions with pathogens [4], humans do not appear to be psychologically prepared to act in consequence [5]. Our modern ecology (dense urban areas where isolation has become a costly good) has made the situation even worse: the virus is continuing to spread and the death toll has recently reached 2 million deaths in the world (https://covid19.who.int).

Governments from all over the world were required to respond quickly and efficiently to the crisis in order to minimize the negative consequences on the current and future health of their population. Consequently, governments had to push very difficult lockdown measures and require its citizens to adhere to these measures in order to stop the spread of the virus. An important number of researches has been conducted on the different measures implemented by governments (especially lockdown and social distancing measures [6–10]). Research have focused on identifying the factors explaining the adherence or resistance to these measures, such as national identification [11], fake news [12], conspiracy theories [13], or personality traits [14]. In other words, numerous studies aimed to understand the behaviors of populations in this crisis context, and to give meaning to the adoption or rejection of government measures.

In this paper, we examine the specific role of perceived threat and how change of values during the COVID-19 pandemic may favor the adherence to government measures. Based on the current scientific literature, we theorize that the endorsement of conservation values during the COVID-19 pandemic would mediate the relationship between perceived threat and adherence to government measures.

### The COVID-19 threat and change of values

Researchers have argued that values refer to goals that serve as guiding principles in people’s life [15, 16]. Schwartz distinguished ten distinct basic motivational human value orientations. These values are clustered into four value domains [17]. Self-transcendence (universalism, benevolence) refers to the tendency to transcend self-interest to promote the well-being of others. On the contrary, self-enhancement (power, achievement) refers to the tendency to favor personal interests to the detriment of those of others. Conservation (tradition, conformity, security) consists in favoring stability and preserving traditional practices. Finally, openness to change (hedonism, stimulation, self-direction) is characterized by an orientation towards change and independence.

If millions of dollars have been invested in research to find and test a vaccine or medication that would stop the contagion or cure the consequences on individuals’ physical health [18], an important amount of research has also focused on the psychological consequences of the COVID-19 crisis [6, 19]. The COVID-19 pandemic is an extraordinary event that provided an opportunity to study whether adverse external factors influence what individuals consider important and worthwhile in life (i.e., values [15, 20]. In particular, how changes in terms of values may influence adherence (or not) to measures represent an important question to better understand how difficult measures may be implemented when an important threat is perceived such as a deadly virus.

If values have been initially theorized as stable and context-free [21], several contributions suggest that values changes reflect external circumstances and important context changes [22–25]. The COVID-19 pandemic and lockdown restrictions implemented in many countries may be considered to constitute such threatening circumstances [26].

The ‘behavioral immune system’ refers to an immune system—besides the physiological immune system that fights pathogens inside the organism—that aims to monitor, detect and avoid physical contact with pathogens [27]. Pathogen prevalence is associated with more social conservatism (strong adherence to norms and traditions [28, 29], a reaction that could be linked to Schwartz’s conservation values. Moreover, under pathogen threat, individuals tend to display increased risk aversion and decreased openness to change [30]. This system thus tends to ‘*favor behaviors that reduce interpersonal contacts—hence limit one’s likelihood of contracting infectious diseases’* [31]. Thus, the behavioral immune system has been found to be associated with concern and preventative health behaviors in the time of COVID-19 [32]. In line with these considerations, the COVID-19 outbreak could be linked with stronger conservation values, and decreased openness to change. This prediction is partly supported by Bojanowska et al.’s [26] results. In a Polish context, they found changes in values due to the COVID-19 pandemic. More precisely, they observed an increase in the importance of conformity and security (two values linked to conservation) and a decrease in that of self-direction (a value linked to openness to change). This relationship between threat and conservation values was also observed in the context of other kind of threats such as the 2008 global financial crisis [33], and previous studies suggested that dangerous world beliefs (i.e., the perceptions of the world as dangerous and threatening) can lead to conservative and authoritarian attitudes [34]).

The first goal of this paper is to compare values and see how they have changed in times of the COVID-19. Based on the predictions of the behavioral immune system, and Bojanowska et al.’s [26] results, we hypothesized that values will tend to conservatism and less to openness to change. Consequently, investigating changes in values due to the COVID-19 outbreak in a different cultural context (French context) and with different methods, constitutes an additional test of the predictions derived from the behavioral immune system and would allow for a better understanding of value changes in times of crisis. Such an understanding is essential in guiding decisions about health-related behavioral and environmental restrictions, not only during the COVID-19 pandemic, but also for future similar crises [26]. In addition, it would contribute to a better understanding of the reactions of populations facing a health crisis such as that of COVID-19.

### The relationships between perceived threat and adherence to government measures: The mediating role of value change

Extensive research has also been conducted in order to raise awareness of the risk among populations, promote the adoption of behaviors that will help prevent the spread of the virus (e.g., social distancing) and, more broadly, promote the adherence to governmental directives. In this regard, one of the most promising solutions is probably ‘psychological’ or ‘behavioral’ [35], and particularly social [10]. Making these measures work and gaining public acceptance is a major challenge for governments [36]. It is therefore critical to identify the factors that influence people’s perception of the threat linked to the COVID-19 pandemic (e.g., threat perception, conspiracy beliefs) and people’s behaviors regarding governments’ instructions [37]. In this respect, previous studies showed a positive relationship between perceived threat and compliance to governmental directives [38–40]. In particular here, respecting social distancing is not straightforward, and a number of factors probably play a role in reducing one’s capability to abide by it. Physical constraints probably operate, such that some parts of our environment (like grocery shops) are not simply made for distant interactions and for operating in circumstances imposed by social distancing. Other constraints are ‘psychological’. They include, for instance, our basic need for social and physical contact, in particular with loved ones, a behavior which probably is reinforced in times of danger [5, 31]. They also include what others do, and the social influences close others have on their own [10].

Cultural factors would also play a key role in these perceptions and behaviors [16, 37]. Indeed, cultural values have been found to guide intentional behaviors, principles in people’s lives. They motivate people to engage in practices that are consistent with their values and to avoid practices that are contrary to these values [15, 41–43]. For instance, individualism has been found to negatively predict intentions to engage in social distancing, while collectivism positively predicted these intentions [37]. More related to Schwartz values, Wolf et al. [44] suggest that compatibility of these values with existing COVID-19 guidelines is probably primordial in shaping our responses to their announcement. Moreover, previous works linked higher conservation values (and lower openness values) to compliance and security behaviors [44–47].

The second goal of the current research is to examine the specific role of cultural values, and specifically conservation, as an explanatory variable between the perception of an important threat in times of a crisis and how the population adheres or not to restrictive governmental measures. We hypothesized that the perception of the severity of the threat will be positively associated with the importance given to conservation values. This importance of conservation values will in turn be positively associated with adherence to government measures. In other words, we put forward the argument that conservation values represent one of the key elements explaining population’s adherence to government measures in times of pandemic.

## Method

### Participants

An online questionnaire was administered to a heterogeneous sample of French citizens during the French lockdown related to the outbreak. The global sample consisted of 1025 participants (21.72% male, 1.24% other, *Mage* = 35.47, *SD* = 15.84). 31.98% were students, 21.39% were senior managers and professional occupations (excluding health services), 17.34% were employees (excluding health services), 7.89% were professionals of the health services, 3.22% were social workers, 2.08% were craftsmen or shopkeepers, 1.66% were factory workers, 0.73% were farmers, 13.71% “other”. 0.52% had no degree, 4.65% before BAC, 21.92% were BAC, 36.92% were BAC+3, 26.58% were BAC+5, and 9.41% were BAC+8 (BAC refers to the French baccalaureate; French baccalaureate). This study was approved by the ethical committee of Clermont Auvergne University (IRB00011540-2020-35). All data can be openly accessed at: https://osf.io/wfsv2/?view_only=db700c5432b743ee9d69ffeefb9209a3

### Materials and procedure

Participants were invited to complete an online questionnaire during the first French lockdown period related to the covid-19 pandemic (between April 02/2020 and May 11/2020). Participants were recruited via posts on social media through the snowball method. After a brief introduction section including a consent form, participants were invited to complete a series of measures.

#### Sociodemographic variables

Participants were first asked to indicate their gender, age, socio-economic status and education level.

#### Values usually in life

Participants’ general values in usual life were measured using the short version of the Portrait Values Questionnaire (PVQ-21 [48]). Participants were presented 21 verbal portraits. Each portrait describes a person and his/her objectives or aspirations, reflecting the importance of a specific value (e.g., “*It is important to him/her to be rich*. *He/she wants to have a lot of money and expensive things*” for the self-enhancement value). For each portrait, participants were asked to report how similar the person is to them in their usual life using a visual analogue scale (from 0 “*Not like me at all”* to 100 “*Very much like me”*). The PVQ-21 allows to measure the ten Schwartz’s motivational value orientations. These measures had adequate internal consistencies (see Table 1).

**Table 1:** Descriptives of the variables (N, missing data, mean, SD, Skewness, Kurtosis and Cronbach alpha).

	N	Missing	Mean	SD	Skewness	Kurtosis	Cronbach alpha
*Values Usually in Life*							
Self-Transcendence	873	152	81.1	12.9	-1.14	2.96	0.67
Conservation	851	174	50.6	17.9	-0.12	-0.30	0.71
Self-Enhancement	873	152	36.5	21.5	0.27	-0.63	0.80
Openness to Change	873	152	68.1	15.5	-0.46	0.67	0.70
*Values during Covid-19 Pandemic*							
Self-Transcendence	740	285	82.3	14.2	-1.28	3.11	0.66
Conservation	730	295	61.0	19.2	-0.71	0.36	0.74
Self-Enhancement	740	285	22.5	20.9	1.05	0.50	0.81
Openness to Change	740	285	57.8	20.1	-0.20	-0.20	0.75
Perceived threat	925	100	67.6	21.2	-0.71	0.39	0.78
Compliance	716	309	94.4	12.4	-3.75	17	-
z-score compliance EXP_transformation	716	309	0.00	1.00	-0.58	-1.64	-
Social distancing	710	315	92.1	17.5	-3.49	13.3	0.71
z-score social distancing_ EXP_transformation	710	315	0.00	1.00	-0.21	-1.92	-

Note:

**p < .01,

***p < .001.

#### Values during Covid-19 pandemic French lockdown

Participants’ values at the current moment, since deaths from covid-19 had first been reported in France, were measured using the same scale as for general values in life (PVQ-21). This time, participants were asked to indicate their similarity to the person described at the current moment, since the coronavirus hit France using visual analogue scales (from 0 “*Not like me at all”* to 100 “*Very much like me”*). These measures had adequate internal consistencies (see Table 1).

#### Perceived threat linked to covid-19

The questionnaire also included a 3-item measure of perceived threat linked to covid-19 (i.e., "*I feel threatened for myself and my loved ones by covid-19*", "*I find covid-19 dangerous*", "*I find the covid-19 crisis to be serious*"). Participants reported their answers using visual analogue scales (from 0 = “*Not at all”* to 100 “*Yes*, *very much*”). This measure presented a good internal consistency (α = 0.78; see Table 1).

#### Social distancing

The questionnaire included a two-item measure of the frequency of adoption of behaviors related to social distancing (i.e., "*I respect a minimum distance of one meter between me and other people when I leave my home*", "*I greet people without shaking their hands and I don’t hug*"). Participants reported their answers using visual analogue scales (from 0 = “*Never”* to 100 “*All the time*”). This measure presented an adequate internal consistency (α = 0.71; see Table 1).

#### Compliance with movement restrictions

The questionnaire included a single-item measure of compliance with movement restrictions (« *Since the beginning of lockdown*, *to what extent have you complied with the following instruction*: *‘Do not leave your home outside the framework set by the government’s current override movement attestation’*”?). Participants reported their answers using a visual analogue scale (from 0 “*No*, *I don’t comply with it at all*” to 100 “*Yes*, *I totally comply with it*”).

## Results

### Preliminary analysis

For each variable, Table 1 presents the sample, the missing data, the mean and the standard deviation, Skewness and Kurtosis, and Cronbach alpha. Because both compliance and social distancing variables were highly skewed and leptokurtic (kurtosis > 3), we transformed them using an exponential transformation. We used the z-scores of the transformed data of these two scales in the statistical analyses.

### The COVID-19 threat and change of values: Values according to the response context

**Fig 1:**
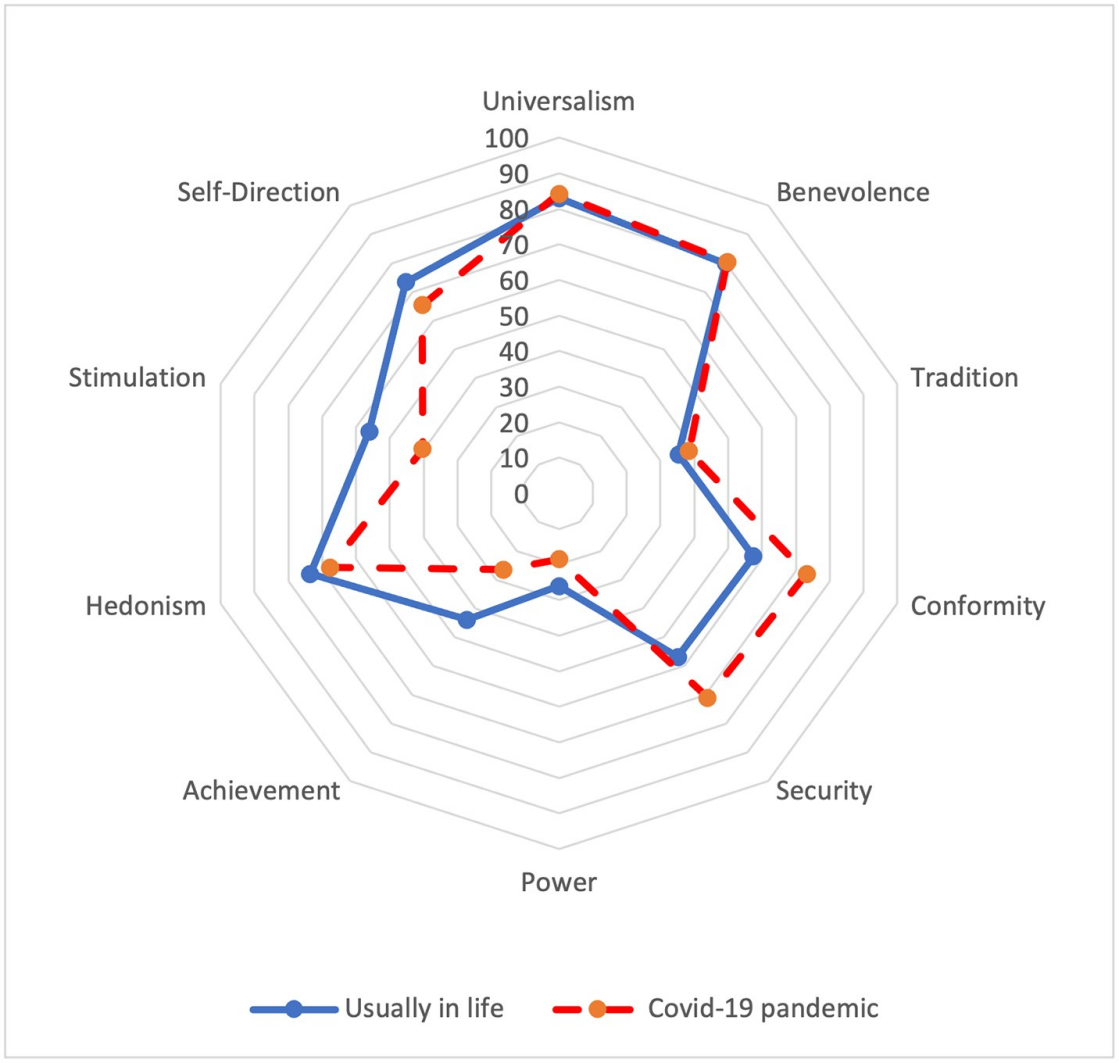
Magnitude of endorsement of Schwartz’ values (0: Not at all; 100: Completely, for each values) according to the response context (i.e., usually in life and during the Covid-19 pandemic).

First, in a series of paired samples t-tests, we compared the endorsement of Schwartz’s values expressed during the Covid-19 pandemic with endorsement scores in the usual living context. For each comparison, we reported in Table 2 the *p*-value and BF_10_ (i.e., the extent to which the data support H_1_). Substantial support for H_1_ was provided by a BF_10_ > 3 (BF_10_ > 10 was judged strong; > 30 very strong and > 100 decisive), and support for H_0_ was provided by a BF_10_ < 1 [49]. Fig 1 illustrates the mean scores of the Schwartz values in both contexts (pandemic vs. usual context). Most of the values were significantly different in the two contexts. We obtained very strong support for greater levels of conservation values and for lower levels of self-enhancement and openness-to-change values in the pandemic context than in the usual context (all *p’*s < .001, and all BF_10_ > 150).

**Table 2:** Means, difference scores and BF_10_ of Schwartz’ values according to the response context (i.e., usually in life vs. during the Covid-19 outbreak).

	Usually in life	Covid-19 outbreak	M_diff_	d	BF_10_
*Self-Transcendence*	81.28	82.27	+0.98**	0.11	2.86
Universalism	82.98	84.23	+1.24***	0.12	8.76
Benevolence	79.83	80.41	+0.57	0.05	0.09
*Conservation*	49.86	61.03	+11.17***	0.92	> 150
Tradition	35.34	38.46	+3.13***	0.22	> 150
Conformity	57.44	73.25	+15.81***	0.82	> 150
Security	56.81	71.10	+14.28***	0.78	> 150
*Self-Enhancement*	35.03	22.54	-12.48***	-0.88	> 150
Power	26.07	18.50	-7.57***	-0.52	> 150
Achievement	43.99	26.59	-17.40***	-0.89	> 150
*Openness to Change*	67.58	57.76	-9.82***	-0.62	> 150
Hedonism	73.48	67.58	-5.90***	-0.30	> 150
Stimulation	56.09	40.33	-15.76***	-0.71	> 150
Self-Direction	73.33	65.40	-7.94***	-0.41	> 150

Note:

**p < .01,

***p < .001.

### Relationships between Schwartz’s values and compliance with movement restrictions

We conducted a series of regression analyses to examine the relationships between Schwartz’s values and compliance with movement restrictions. First, we computed the standardized beta coefficient between each value and the dependent variables. As depicted in Table 3, self-transcendence and conservation values, whatever the context (i.e., usual and during the outbreak), were significantly and positively related to compliance. Then, we conducted a multiple regression analysis with the values that were significantly related to compliance. We also added sociodemographic variables (i.e., age, gender, socioeconomic status, and education). All VIFs were inferior to 3, indicating an absence of multicollinearity. As shown in Table 3, controlling for significant values and for sociodemographic variables, two values still remained significantly related to compliance: self-transcendence in the usual life context (*ß* = 0.15, *p* < .01, BF_10_ = 18.3), and conservation in the context of the outbreak (*ß* = 0.22, *p* < .001, BF_10_ = 552.8). Education was modestly but significantly related to compliance with movement restrictions (*ß* = 0.09, *p* < .05, BF_10_ = 0.94). The more the participants were poorly educated, the less they complied with movement restrictions.

**Table 3:** Beta coefficients and partial coefficients (standardized) between Schwart’s values, compliance with movement restrictions and social distancing.

	Z-score Compliance (EXP_transformation)			Z-score Social distancing (EXP_transformation)		
	*ß*	*Partial ß*	VIF	*ß*	*Partial ß*	VIF
*Values Usually in Life*						
Self-Transcendence	0.16***	0.15**	2.49	0.11**	0.01	2.53
Conservation	0.15***	-0.03	2.73	0.12**	0.01	2.97
Self-Enhancement	-0.06	-	-	-0.07	-	-
Openness to Change	-0.05	-	-	0.00	-	-
*Values during Covid-19 Pandemic*						
Self-Transcendence	0.14***	-0.03	2.58	0.12***	0.07	2.59
Conservation	0.21***	0.22***	2.86	0.16***	0.14*	2.89
Self-Enhancement	-0.06	-	-	-0.09*	-0.10*	1.25
Openness to Change	-0.06	-	-	-0.02	-	-
Age	-	-0.03	1.15	-	0.05	1.23
Gender	-	-0.05	1.03	-	-0.04	1.07
SES	-	0.05	1.38	-	-0.05	1.38
Education	-	0.09*	1.27	-	-0.05	1.27

Note:

***p < .001,

**p < .01,

*p < .05.

VIF = Variance Inflation Factor. Partial *ß* were controlled for values that were significantly related to the outcome and for socio-demographic variables (i.e., age, gender, SES, and education).

### Relationships between Schwartz’s values and social distancing

We replicated the same statistical procedure with the measure of social distancing as a dependent variable. Beta coefficients indicated that several values were significantly related to social distancing (see Table 3). However, a multiple regression analysis (all VIFs < 3) revealed that only two values were robustly related to social distancing: conservation and self-enhancement during the outbreak (respectively, *ß* = 0.14, *p* < .05, BF_10_ = 19.7 and *ß* = -0.10, *p* < .05, BF_10_ = 8.3). Sociodemographic variables were not related to social distancing.

### Relationships between perceived threat, cultural values, compliance with movement restrictions and social distancing

Table 4 presents relationships between perceived threat and Schwartz’s values. Beta coefficients indicated that except for usual openness to change and self-enhancement during the outbreak, all the other values were significantly related to perceived threat. A multiple regression analysis (all VIFs < 3) revealed that only two values were robustly related to perceived threat: conservation and openness to change during the outbreak (respectively, *ß* = 0.35, *p* < .001, BF_10_ = 3.2e+8 and *ß* = -0.20, *p* < .001, BF_10_ = 1.75e5). Sociodemographic variables were not related to perceived threat.

**Table 4:** Beta coefficients and partial coefficients (standardized) between perceived threat and Schwart’s values.

	Perceived Threat		
	*ß*	*Partial ß*	VIF
*Values Usually in Life*			
Self-Transcendence	0.12***	0.00	2.59
Conservation	0.32***	0.00	2.95
Self-Enhancement	0.09**	0.04	1.37
Openness to Change	-0.02	-	-
*Values during Covid-19 Pandemic*			
Self-Transcendence	0.13***	0.10	2.81
Conservation	0.41***	0.35***	2.94
Self-Enhancement	-0.01	-	-
Openness to Change	-0.20***	-0.20***	1.23
Age		0.05	1.30
Gender		-0.03	1.07
SES		0.03	1.39
Education		-0.04	1.28

Note:

***p < .001,

**p < .01,

*p < .05.

VIF = Variance Inflation Factor. Partial *ß* were controlled for values that were significantly related to the outcome and for socio-demographic variables (i.e., age, gender, SES, and education).

Finally, we examined the relationships between perceived threat, compliance with movement restrictions and social distancing. Perceived threat was positively and significantly related to both compliance with movement restrictions and social distancing (respectively, *ß* = 0.19, *p* < .001, BF_10_ = 1.75e4 and *ß* = 0.21, *p* < .001, BF_10_ = 3.78e5). Compliance with movement restrictions and social distancing also were positively and significantly correlated (*r* = .25, *p* < .001).

### The relationships between perceived threat and compliance with movement restrictions (A) and social distancing (B): The mediating role of value change

We tested our two predicted mediation models (i.e., model A: perceived threat -> conservation during Covid-19 pandemic -> compliance with movement restrictions; model B: perceived threat -> conservation during Covid-19 pandemic -> social distancing) using GLM Mediation Analysis provided by Jamovi 1.2.27.0. We selected the following specifications: 5000 bootstrap (BC) samples and 95% confidence intervals.

The first two requirements for the mediation procedure are that the independent variable (i.e., perceived threat) be related to the dependent variable (i.e., compliance for model A and social distancing for model B) and the mediator (i.e., conservation during Covid-19 pandemic), which was the case (see previous section). In addition, the mediating variable (i.e. conservation during Covid-19 outbreak) should be significantly related to the dependent variable (i.e., compliance for model A and social distancing for model B), which was also the case (see Table 3). Fourth, the mediating variable should predict the dependent variable, even when the independent variable is statistically controlled, while the effect of the independent variable on the dependent measure should be significantly reduced when the mediating variable is statistically controlled. We tested this step sequentially for each dependent variable.

When conservation during Covid-19 outbreak was statistically controlled for, the relationship between perceived threat and compliance with movement restrictions remained significant (direct effect: *ß* = 0.12, *z* = 2.94, *p* < .003; b = .006, se = .002; 95%CI [.002; .009]), but was significantly reduced (indirect effect: *ß* = 0.07, *z* = 3.65, *p* < .001; b = .003, se = 8.64e-4; 95%CI [.002; .005]). Thus, conservation during Covid-19 pandemic partially mediated the relationship between perceived threat and compliance with movement restrictions (see Table 5, model A).

**Table 5:** Path-analysis models showing the direct, indirect, and total effect of perceived threat on compliance with movement restrictions (model A) and social distancing (model B) via endorsement of conservation during Covid-19 pandemic.

Effect	Estimate	SE	95% C.I. (BC)		*ß*	z	p
			Lower	Upper			
Model A: Perceived threat -> Conservation during outbreak -> Compliance							
Indirect	.003	8.64e-4	.002	.005	.07	3.65	< .001
Direct	.006	.002	.002	.009	.12	2.94	.003
Total	.009	.002	.005	.012	.19	5.04	< .001
Model B: Perceived threat -> Conservation during outbreak -> Social Distancing							
Indirect	.002	8.37e-4	2.79e-4	.004	.04	2.19	.029
Direct	.008	.002	.004	.01	.17	4.16	< .001
Total	.01	.002	.006	.01	.21	5.65	< .001

When conservation during Covid-19 outbreak was statistically controlled for, the relationship between perceived threat and social distancing remained significant (direct effect: *ß* = 0.17, *z* = 4.16, *p* < .001; b = .008, se = .002; 95%CI: [.004; .01]), but was significantly reduced (indirect effect: *ß* = 0.04, *z* = 2.19, *p* < .029; b = .002, se = 8.37e-4; 95%CI: [2.79e-4; .004]). Thus, conservation during Covid-19 pandemic partially mediated the relationship between perceived threat and social distancing (see Table 5, model B).

## Discussion

The present study first aimed at evaluating a possible relationship between the COVID-19 pandemic and people’s values in a sample of the French population. As predicted, the perceived threat linked to COVID-19 was related to an increase in conservation values (i.e., favoring stability, conformity, security, preserving traditional practices). This result is consistent with the predictions of the behavioral immune system according to which pathogen threats such as COVID-19 are associated with more social conservatism (stronger adherence to norms and traditions [28, 29]. This increase in conservation values would thus reflect the reaction consisting in favoring behaviors allowing to avoid contact with pathogens threats [32]. It is also in line with Bojanowska et al.’s [26] results in a Polish context. More precisely, while Bojanowska et al. [26] only observed an increase in the importance of two of the three values clustered into the conservation value domain (conformity and security, but not tradition) due to COVID-19 threat, the present results tend to support an increase in the importance of conservation values as a whole. Furthermore, these results support the idea according to which Schwartz values can change in response to external circumstances and important context changes [24, 25], here the COVID-19 pandemic and lockdown restrictions [26]. Furthermore, as predicted, openness-to-change (hedonism, stimulation, self-direction) values were lower during the Covid-19 outbreak than usual. The same pattern was observed for self-enhancement (power, achievement). These results illustrate the malleability of values (particularly in what appears to be a life-threatening context), and challenge the initial theorization of these values as stable and context-free [21].

The present study also aimed to examine the specific role of conservation values during the COVID-19 pandemic in the relationship between threat perception and adherence to restrictive governmental measures. Our results not only supported the positive effects of perceived threat linked to COVID-19 on both compliance with movement restrictions (model A) and social distancing (model B), but also indicated that both effects were partially mediated by the importance of conservation values during COVID-19. The stronger the threat, the more people tend to favor conservation values and, as a consequence, the more they tend to adhere to government measures. In other words, and consistent with the predictions derived from the behavioral immune system, conservation values appear to be important factors in the relationship between a significant pathogen threat such as COVID-19 and the adherence to instructions aiming to avoid being contaminated and spreading the virus. Thus, cultural factors would play a key role in the adoption of behaviors that help to prevent the spread of COVID-19 [16, 37]. More broadly, this result is consistent with the motivational nature of cultural values (individuals’ guiding principles; [15, 16]. Especially, in the COVID-19 context, conservation values—the tendency to favor tradition, conformity and security—seem to motivate, as a result of threat perception, the adoption of behaviors designed to protect oneself against the virus. This result is consistent with previous works that linked higher conservation values to compliance and security behaviors [44–47]. They are also in line with previous works showing that perceived threat related to COVID-19 tended to increase success for conservative political parties in France [31], but also with the model of political conservatism as motivated social cognition showing that fear of threat and loss positively predict political conservatism [50]. Furthermore, interestingly, Pagliaro et al. [51] found that the actual threat posed by COVID-19 (deaths per million people) did not significantly predict individuals’ compliance with preventive behaviors. Our results thus point to the importance to distinguish perceived (in our study) and actual (Pagliaro et al.’s [51] study) threat when it comes to consider the links between threat and compliance with movement restrictions.

There are, however, some limitations that must be considered regarding the present study. First and foremost, behavioral measures (i.e., compliance with movement restrictions and social distancing) were self-reported measures. If social desirability is not systematically associated with self-reported current health behavior [52], the possible influence of social desirability on our self-reported measures cannot be excluded (in the case of COVID-19 preventive behaviors [53]). Perceived threat and cultural values, however, could only be measured through self-reported measures. Second, this study focused on cultural values using the ten values model of Schwartz. However, other models exist and are widely used, such as the cultural dimensions model [54], and other cultural factors as cultural tightness (severity of social norms and tolerance for deviance [55]) have been found to affect the containment of COVID-19 [56, 57]. Furthermore, the small size of the indirect effects observed in our mediation analyses suggests that other mediating variables should be considered in future studies. Third, we tested our hypotheses in a specific national and cultural context—the French context—what limits the generalizability of the present results. However, it is worth noting that the malleability of cultural values due to the COVID-19 pandemic has also been observed in a Polish context [26]. Further research could be conducted from other countries to determine whether the impact of Covid-19 crisis on individual’s endorsement of cultural values is similar to our study.

Our study focused on the difference between people’s endorsement of Schwartz’s values in usual life and people’s endorsement of Schwartz’s values during the Covid-19 pandemic’s first lockdown. From our results, we can infer that cultural values, to a certain degree, are context-dependent. Indeed, shifts in the importance placed on cultural values do occur during the first lockdown, supporting the idea that values are adaptations to the environment [22–25]. People coped with the Covid-19 pandemic threat in increasing behaviors that are motivated by conservation values (i.e, values that help to avoid the threat of uncertainty and for which primary motivational goals are safety and stability [15]). However, there is some evidence that rebound effects appear with time elapsing after a major life transition or a traumatic event [58, 59]. Personal values appear to react to changing contextual circumstances but then return close to their baseline levels after a more or less long period. Our study didn’t directly test an evolution of the cultural value in time. The question remains then to know whether the effect of the Covid-19 pandemic was a sufficiently powerful influence on the lives of French to change them in the deep levels of their personalities. It would be useful to compare cultural value’s endorsement at different moments of the pandemic crisis (i.e., before, during and after).

Several factors could explain that the changes could be stable or diminish over the time. One of them is the evolution of the perceived threat linked to COVID-19. Since the first lockdown, several information about what is the coronavirus and how to protect oneself from it and avoid its spread has been communicated by social media and French authorities. These knowledge may have maintained people’s feeling of threat toward the virus, and thus a high level of conservation tendencies. On the contrary, it may have allowed people to gain confidence that they get control over the coronavirus and thus decrease the feeling of threat associated with the coronavirus. In the same time, the government’s multiplication of recommendation and constraints, the go and back between periods of lockdown and curfew, the duration of the pandemic crisis and the social isolation in which it plunged people, may have given place to other important values that are opposed to conservation value. Openness to Change values (Self-Direction, Stimulation, Hedonism), emphasize own independent thought and action and favor change. It stimulates actions that promote self-direction values like independence and freedom. Isolation or reactance toward the government’s initiatives may have reinforced this fundamental value. In our study, this value had been perceived by people as less important during the lockdown and as a consequence, did not predict compliance with movement restrictions and social distancing. However, it was negatively linked with perceived threat, suggesting that a possibility, if this value has gained in importance since the first lockdown, is that it may motivate conflicting behavior towards compliance with movement restrictions and social distancing. Researchers therefore should explore how long those changes in cultural values lasts, how much threat exposure is needed to see long-term attitudinal change and whether these changes also impact other kinds of public health related behaviors.

## Conclusion

In this study, the impact of Schwartz’s four high order values (i.e., conservation, self-enhancement, self-transcendence and openness to change) on compliance with movement restrictions and social distancing was examined. It provides empirical evidence that individuals are guided by particular values that influence their attitudes and behaviors. In times of crisis, such as during the pandemic COVID-19 pandemic, value conservation is a significant predictor of the compliance with movement restrictions and social distancing. People concentrate on themselves, and values related to health and economic security become more important. That is because a lot of people perceive the COVID-19 pandemic as a threat. Our study underlines that contextual variables are important to understand value priorities and their potential changes over the time.
